# Preventing and Managing Radial Artery Occlusion following Transradial Procedures: Strategies and Considerations

**DOI:** 10.3390/jcdd10070283

**Published:** 2023-06-30

**Authors:** Grigorios Tsigkas, Amalia Papanikolaou, Anastasios Apostolos, Angelos Kramvis, Filippos Timpilis, Anastasia Latta, Michail I. Papafaklis, Adel Aminian, Periklis Davlouros

**Affiliations:** 1 Department of Cardiology, University Hospital of Patras, 265 04 Patras, Greece; amaliapap22@gmail.com (A.P.);; 2 First Department of Cardiology, Hippocration General Hospital, National and Kapodistrian University of Athens, 157 72 Athens, Greece; 3 Department of Cardiology, Centre Hospitalier Universitaire de Charleroi, 6042 Charleroi, Belgium

**Keywords:** RAO, radial artery occlusion, dTRA

## Abstract

Τransradial artery access has recently gained widespread acceptance as the preferred approach for coronary angiography and interventions, due to its lower incidence of bleeding and vascular complications compared to transfemoral access. However, thrombotic occlusion of the radial artery has emerged as the most common complication of this method, impeding its use in future interventions, and in the creation of arteriovenous fistulae for hemodialysis patients, or as a graft for coronary artery bypasses grafting. In this comprehensive review, we delve into the anatomy of the radial artery, the pathophysiology and diagnosis of radial artery occlusion, the identification of potential risk factors and, finally, prevention and treatment strategies. We acknowledge that distal transradial access provides an effective alternative for coronary angiography and catheterizations, with a reduced incidence of radial artery occlusion.

## 1. Introduction

Over the past few decades, percutaneous coronary angiography and interventions have gained prominence in the diagnosis and treatment of coronary artery disease. Traditionally, femoral artery access was favored, due to its larger diameter, easier puncture, and catheter engagement of coronary arteries. However, the increased incidence of local complications, bleeding, and prolonged immobilization of patients, prompted the search for alternative access routes through the arm. Although attempts to use the brachial and ulnar arteries as access points failed to gain widespread clinical acceptance, transradial artery access has emerged as a viable alternative. Transradial artery access is currently considered to be the primary approach for coronary angiography and percutaneous coronary interventions, due to fewer vascular complications, reduced all-cause mortality, and major bleeding events. Moreover, it offers quick mobilization to the patient and earlier discharge from the hospital, compared to the brachial and femoral artery approach [1].

**Figure 1:**
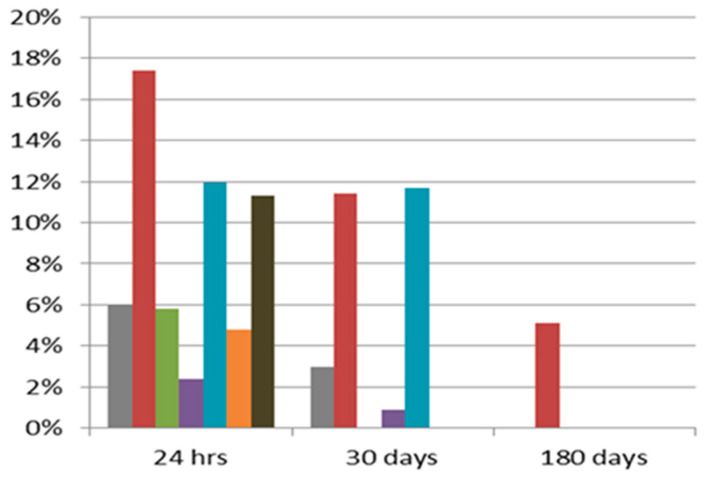
RAO incidence according to timing of assessment. (each color represents the results of each trial) da Silva 2022 [3]; Dwivendi 2022 [4]; Mohsen 2018 [5]; Munir 2022; [6]; Ognegurov 2020 [7]; Pacchioni 2019 [8]; and Sinha 2017 [9].

Similar to other interventional procedures, transradial coronary catheterization is occasionally associated with various complications, such as radial artery occlusion (RAO), forearm hematoma, pseudoaneurysms, arteriovenous fistula formation, compartment syndrome, radial artery perforation, nerve damage, and local infections [2]. RAO appears to be the most common complication, with an incidence ranging from 1% to 30%, according to the timing of assessment (Figure 1).

The aim of this comprehensive manuscript is to provide an in-depth review of radial artery anatomy, pathophysiology of RAO, diagnostic methods, risk factors and preventive strategies, the efficacy of distal transradial artery access, and treatment options for RAO [3,4,5,6,7,8,9].

## 2. Anatomy of the Radial Artery

The radial artery is one of the major supplying arteries of the upper limb, and it consists of three portions: the first in the forearm, the second in the wrist, and the third in the hand palm.

In the majority of people, its proximal part starts at the bifurcation of the brachial artery at the superior border of the cubital fossa, a triangular area defined by the lateral border of the pronator teres, the median border of the brachioradialis, and an imaginary border between the epicondyles. Its medial part runs through the radial side of the forearm, between the brachioradialis and the flexor carpal radialis and, at last, it turns laterally at the styloid process of the radius, where the superficial palmar branch arises, and ends up at the distal part of the artery, in the anatomical “snuffbox”, an anatomical area of the hand defined by the three muscles that act on the thumb: the abductor pollicis longus, the extensor pollicis brevis, and the extensor pollicis longus. Finally, it passes forward, between the two heads of the first interosseous dorsalis muscle, into the palm of the hand, where it crosses the metacarpal bones and, at the ulnar side of the hand, unites with the ulnar artery to form the deep palmar arch.

Due to existence of the deep and the superficial palmar arches, the radial artery has, in most people, a satisfactory ulnar collateral supply; however, it should be stated that the superficial palmar arch appears to have, in a minority of cases, variations with a clinical significance. Unlike the brachial and the femoral artery, the radial artery is not considered to be an end artery; hence, in most patients, its occlusion does not compromise the vascular supply to the hand [10,11].

## 3. RAO Pathophysiology

RAO may arise typically due to endothelial injury and decreased blood flow during the insertion of sheaths and catheters at the catheterization site, as well as during manipulations throughout the procedure. More specifically, during sheath insertion, the intima of the vessel may be damaged, leading to local exposure of collagen fibers and tissue factors, while blood flow is halted in the radial artery. Consequently, the coagulation cascade is triggered, causing platelets and fibrin to aggregate at the site of vascular injury, leading to thrombus formation and local thrombosis of the radial artery.

However, arterial occlusion following percutaneous coronary procedures could be a result of compression phenomena, due to local induction by the catheterization process edema or hematoma [12,13].

The dual blood supply to the hand confers a quiescent character of RAO in most patients, which results in the majority of cases going undetected. Symptoms indicating RAO, such as pain at the site of the thrombosis, paresthesia, and decreased limb function, are only present in a minority of cases, accounting for approximately 0.2% of patients. Hand ischemia due to RAO occurs exclusively in individuals with insufficient collateral circulation, mostly on account of anatomic variations [14].

## 4. RAO Diagnosis

Doppler ultrasonography is widely considered to be the gold standard method for assessing radial artery patency following transradial access, owing to its ability to provide both hemodynamic information and anatomical details of the vessel. Nevertheless, current research has investigated alternative methods, taking into account factors such as cost, time, and availability, in order to assess their efficacy in detecting RAO.

Digital plethysmography, using a modified reverse Barbeau’s test, is a more affordable and less time-consuming method compared to a Doppler ultrasound. Plethysmography sampling involves placing a digital sensor on the index finger or thumb to evaluate the patency of the radial artery after transradial access. This is achieved by compressing both the radial and ulnar arteries, causing the oximetry signal to be lost. The release of the radial artery leads to the recovery of the signal, indicating radial artery patency.

While the modified reverse Barbeau’s test, similar to the modified Allen’s test, has been, until now, used as a method to clinically assess the adequacy of the ulnar artery’s collateral flow before the radial artery’s catheterization, in recent randomized controlled trials (RCTs), the effectiveness of this method has been compared to that of Doppler ultrasonography (USG) in detecting RAO. Important findings indicate that there is no significant difference in the efficacy of the two methods [14,15,16].

Laser perfusion imaging (LPI) has recently emerged as a novel and operator-independent diagnostic tool to evaluate radial artery perfusion following transradial catheterization. This diagnostic method involves capturing a color-coded perfusion image of the hand palm in a state of rest. Subsequently, a second image is captured after occluding the ulnar artery with pressure. The diagnosis of RAO is determined by calculating the radial perfusion index (RPI), which is the ratio between the basal radial perfusion and the value measured under ulnar occlusion. Notably, LPI is particularly useful for patients with anatomical variations presenting difficulties during catheterization. Furthermore, the lack of direct skin contact during LPI eliminates any pain or infection associated with probe contact. Additionally, the operator-independent nature of LPI makes it more objective and cost-effective than other methods. Although LPI is a relatively new technology, its potential as a reliable and practical diagnostic tool for RAO warrants further investigation [17].

## 5. RAO Inducing Factors

Many factors contribute mainly to thrombus formation and, consequently, predisposition to RAO. These can be divided into modifiable and non-modifiable, or patient-related and procedurally-induced risk factors (Table 1).

Several patient-related risk factors have been identified for RAO, including younger age, female sex, low body mass index, diabetes mellitus, dyslipidemia, peripheral artery disease, multiple vessel coronary artery disease, reduced renal function, and previous radial artery cannulation. Among these factors, cardiovascular risk factors could be modified over the years through lifestyle changes and pharmacological interventions, whereas previous radial artery cannulation is an unmodifiable risk factor that does not constitute a generic characteristic. Unfortunately, repeated radial cannulation leads to intimal hyperplasia and intima-media thickening, resulting in reduced lumen diameter [18].

On the other hand, hypertension has been proposed to exert a protective effect against RAO, by inducing a hyperdynamic state within the arterial lumen, leading to gradual reopening of the occluded portion of the artery. Consequently, unlike other known risk factors for atherosclerosis, hypertension appears to have a unique effect in this regard [19].

On top of that, there are some procedural RAO inducing factors, which could be mitigated by the operator’s experience and focus during the operation on minimizing traumatic injury to the radial artery. These risk factors include a sheath-to-artery ratio greater than 1, repeated unsuccessful attempts at a radial artery puncture, a radial artery spasm, the use of multiple catheters, periprocedural anticoagulation, occlusive hemostasis and, finally, longer periprocedural and hemostasis times.

Post-procedural pain at the cannulation site and hematoma formation are both considered to be contributing factors to thrombotic radial artery occlusion. Paradoxically, the formation of a hematoma after removal of the hemostatic device is thought to increase the risk of thrombus formation, as the operator may need to manipulate the access site in order to minimize its extent [18,20].

The minimization of the modifiable risk factors could be a helpful tool for reducing rates of post-procedural radial artery thrombosis [6].

## 6. RAO Preventive Strategies

i. Repeated radial punctures and Doppler evaluation

A recurrent endothelial injury from repeated punctures can lead to damage of the vessel wall and early thrombus formation. Thus, the pre-interventional visualization of the radial artery in both limbs using Doppler ultrasonography is suggested in order to assess the radial artery’s characteristics. More specifically, it is useful for the selection of an artery with a wider diameter, and the illustration of the caliber and depth of the vessel. As a result, the cannulation of the artery is more easily achieved, and the need for repeated puncture attempts is reduced [28,29]. Additionally, this imaging modality can aid in selecting an appropriate sheath size to reduce the sheath-to-artery mismatch, thereby minimizing the risk of RAO [3].

ii. Sheath Size

Minimizing the size of equipment used in coronary interventions is crucial in reducing the incidence of RAO. A sheath-to-artery diameter ratio mismatch can result in an injury to the radial artery wall, endothelial dysfunction, blood flow disturbance, and chronic vascular remodeling. Studies have shown that a sheath-to-artery diameter ratio of less than 1 is associated with a lower risk of RAO.

However, this can be difficult to achieve, given the mean inner diameter of the radial artery (approximately 2.5 ± 0.4 mm) and the outer diameter of a 6F sheath (2.52 mm). Complex coronary procedures, such as those involving CTOs, bifurcations, and tortuous calcification lesions, require catheters with larger diameters than 6F, with the outer diameter of a 7F sheath being 3.02 mm. This can result in significant damage to the endothelial wall during these procedures.

Pre-procedural imaging, such as Doppler ultrasonography, may assist in the measurement of the radial artery’s diameter and, thus, the selection of an appropriate sheath size to reduce the sheath-to-artery diameter ratio mismatch [30].

Indeed, data collected from RCTs comparing 5F to 6F RAO rates show lower RAO rates of almost 50% in the 5F groups [31,32].

**Figure 2:**
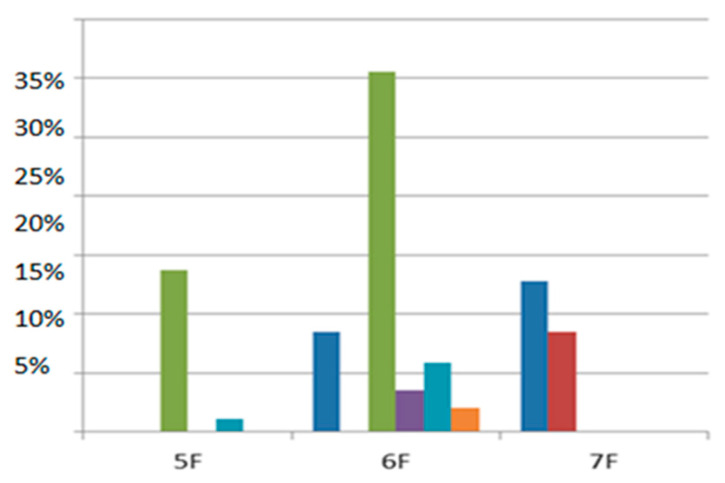
RAO incidence according to sheath size (each color represents the results of each trial). Wang 2021 [21]; Horie 2018 [34]; Aminian 2017 [36]; Dahm 2002 [31]; Uhlemann 2012 [32].

Nevertheless, it has been recently demonstrated that the RAO rate after catheterization with a 7F sheath is less than 10%, and no significant statistical difference in the RAO rate was found between 6F and 7F sheaths [33] (Figure 2). These findings may be attributed to the radial artery diameter variation based on various factors, such as mechanical extension and vasodilatory drug administration.

A new promising entry in the literature is the sheathless transradial access, which can possibly change the sheath-to-artery cutoff ratio for RAO to 1.5 instead of 1 [34,35]. In addition to that, new generation sheaths with more ergonomic characteristics are being developed. For example, the 6 F Glidesheath Slender is a thin-walled radial sheath, with an outer diameter smaller than the one of standard 6 F sheaths, that was studied in the RAP BEAT trial [36].

iii. Vasodilators

It is argued that the subcutaneous injection of nitroglycerin at the radial artery puncture site offers significant vasodilation of the radial artery. This approach facilitates the cannulation. As a result, the number of punctures and the incidence of radial artery spasm are reduced [37,38,39].

Even so, its impact on radial artery occlusion is controversial. Until recently, the pre-hemostasis injection of 500 μg nitroglycerin seemed to reduce the rate of RAO due to the influence of nitrous oxide on the intimal inflammation and hyperplasia of the radial artery [27,37]. However, a recently published study came to overthrow this argument by showing that nitroglycerin had no impact on the incidence of RAO regardless of its administration timing, whether upon sheath insertion or before its removal [3].

Noteworthy is the use of flow-mediated vasodilation (PO-FMD) which is performed by the inflation of a blood pressure cuff for ten minutes prior to the cannulation above the antecubital fossa. Until now, it has been shown to be beneficial regarding the radial artery puncture attempts, the cannulation failure rates, and the cannulation duration for patients with a high risk profile for access difficulties. Nonetheless, its effect on RAO and radial artery spasm (RAS) was no different compared to the traditional methods [40,41].

iv. Anticoagulation

Adequate anticoagulation is an important aspect within the multifactorial preventive strategy for the prevention of RAO, as its pathogenesis is majorly associated with thrombus formation. A bolus dose of 50 IU/Kg or 5000 IU unfractionated heparin (UFH) was, until recently, recommended to be efficient in RAO prevention, regardless of the delivery route, whether intravenously or via the arterial sheath [42]. For this reason, the effect of the standard heparinization dose on RAO has been compared with higher or lower doses in multiple RCTs.

The superiority of the standard over a lower UFH dose of 25 IU/kg on RAO occurrence has been proven multiple times [20,43,44]. Taking this into consideration, recent studies tried to examine whether there is an additional advantage to a high dose of 100 IU/kg UFH. In fact, RAO incidence in the high-dose groups was reduced more than 50% compared with the standard UFH dose, without affecting the bleeding event rates [45,46].

Although higher doses seem to be beneficial for preventing RAO, the optimal heparin dose remains under investigation. One potential resolution for these conflicting findings in the literature is to individualize anticoagulation dosing by measuring the activated clotting time (ACT) for each patient. The use of ACT is crucial, as studies have demonstrated that inadequate or excessive anticoagulation is linked to an increased risk of RAO. Personalizing the anticoagulation dose based on each patient’s ACT measurement could potentially reduce the incidence of RAO following transradial catheterization [8].

Additional research is necessary to evaluate the efficacy of alternative antithrombotic therapies in conjunction with heparin. Recently, the use of prophylactic 10 mg rivaroxaban for seven days after the coronary procedure was compared to placebo in patients undergoing coronary catheterization. While there was no discernible difference in RAO incidence upon discharge, those in the experimental group had significantly lower rates of RAO during one month of follow-up, primarily due to recanalization [26].

v. Hemostasis Methods

The procedure between sheath removal and the removal of the hemostatic device is defined as hemostasis. Occlusive hemostasis, hemostatic devices, and hemostasis duration play a major role in thrombus formation, due to the initiation of decreased blood flow.

Evidence-based contemporary protocols have shown that not only has the option for different hemostatic devices little effect on radial artery thrombosis, but also that the presence of non-occlusive hemostasis appears to be a much more important factor [47,48,49].

Non-occlusive or patent hemostasis is defined by the preservation of antegrade arterial flow, and is achieved by the assistance of the reverse Barbeau’s test. A plethysmography sensor is placed either on the thumb or index finger of the involved upper extremity. Therefore, pulsatile waveforms are observed. The hemostatic compression pressure should be set to the point where an antegrade plethysmography waveform appears during the ulnar artery compression, and at the same time hemostasis is maintained. This return of the waveform is evidence of antegrade radial artery flow [50].

The optimal hemostasis duration is still under investigation. Various research protocols have studied the value of ultrashort, short and long hemostasis durations. It was only proven that ultra-short (20 min) compression of the radial artery results in higher incidence of RAO, as well as hematomas, due to the more frequent retightening of the clamp [51]. Moreover, the same effect is observed in prolonged hemostasis duration protocols (8 h). Although manual compression requires less time for fast hemostasis compared to mechanical, RAO incidence is comparable among the two methods [21,52].

The right deflation timing of the hemostatic band is also of great importance, because it may result in repeated bleeding and re-inflation, factors that contribute to the initiation of the coagulation cascade, favoring RAO appearance. The removal of 3–5 mL of air every 15 min after 120 min of the placement of the device is supposed to be an effective method [53,54].

Although the patent hemostasis technique is currently considered to be the most preferable one, new techniques have been recently developed, and seem to have great potential regarding RAO [55]. A technique where hemostasis is achieved during a simultaneous ulnar and radial compression (SURC) has been lately studied. The SURC technique was not only proven effective, but also superior to the non-occlusive hemostasis one concerning RAO incidence, both at removal time and one month later [24,50,56,57].

## 7. New Approaches: Access through the Distal Part of the Radial

**Figure 3:**
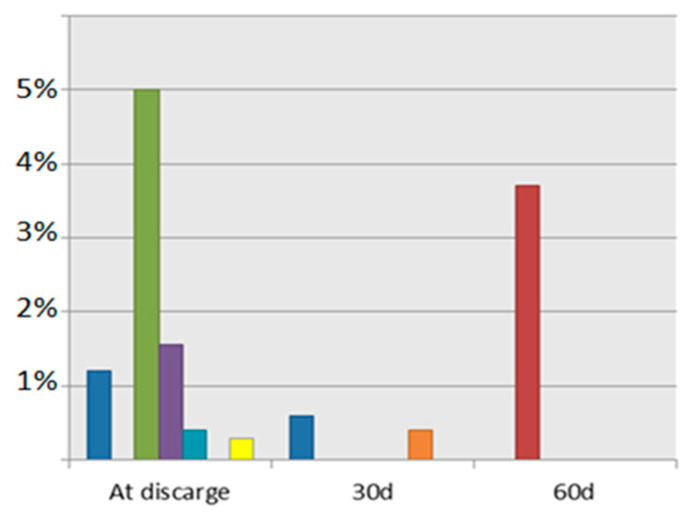
RAO incidence after dTRA (each color represents the results of each trial). Tsigkas 2022 [13]; Aminian 2017 [36]; Mizuguchi 2020 [58]; Lidt 2021 [59]; Koutouzis 2019 [60]; Lin 2020 [61]; Xie 2021 [62].

Despite the implementation of various preventive measures during catheterization, the incidence of RAO remains relatively high. An emerging approach in the field involves accessing the coronary arteries through the distal third of the radial artery (i.e., distal transradial access [dTRA]). Specifically, the catheterization point is situated in the anatomical “snuffbox”, an area characterized by the presence of the abductor pollicis longus and extensor pollicis brevis tendons and the medial tendon of the extensor pollicis longus muscle. This technique shows promise in reducing the incidence of RAO (Figure 3) [11].

As shown in Figure 3, from the RAO rates of some of the largest trials that studied the dTRA approach over the last 5 years, it is obvious that the access through the anatomical snuffbox is superior to the TRA, concerning RAO incidence. RAO incidence after catheterization through the dTRA varies between 0.4% and 5%, once again depending on the assessment timing [13,58,59,60,61,62]. This positive effect on the vascular thrombosis is potentially owing to a shorter hemostasis duration and maintained flow at the wrist during compression. The occlusion rates could be further limited with the use of the proper hemostatic device. For instance, the Prelude Sync Distal radial compression device (PSD) is proved to be superior to the TR-Band in terms of local complications [63].

Furthermore, the access through the distal part may offer an alternative to the transulnar or transfemoral approaches, in cases of an occluded radial artery [64].

Overall, dTRA offers a safe and efficient alternative approach. The wider use of dTRA, especially in cases of cardiovascular emergencies such as ST-segment elevation, myocardial infarction, or cardiogenic shock, is currently under investigation [13,65,66].

## 8. RAO Treatment

Although RAO is suggested to be treated only in symptomatic patients, a patent radial artery is of great importance, because otherwise it could not be used for coronary artery catheterization in the future, for grafting purposes during coronary artery bypass surgeries, and for arteriovenous fistula formation in case of hemodialysis. In addition, prior RAO is a contraindication for the ipsilateral transulnar approach, which further limits the operator’s options in the catheterization laboratory, especially when time is of the essence.

If RAO is diagnosed before discharge, the ipsilateral compression of the ulnar artery for an hour may be an effective technique in order to regain the artery’s patency. This method is associated with an increase in radial blood flow, thereby initiating the release of vasodilator mediators [67].

Apart from this, for patients with persistent RAO, the therapeutic options include either medical treatment with anticoagulation agents for 1–3 months, or percutaneous retrograde revascularization [68,69,70,71].

In most cases, the anticoagulation therapy of choice for people suffering from symptomatic RAO is a body-weight-adjusted dosage of low-molecular-weight-heparin (LMWH) agents. More specifically, in the majority of patients it is either enoxaparin 10 mg per 10 kg body weight, subcutaneously (s.c.) twice daily administered or, for people weighting between 50–100 kg, s.c. fondaparinux 7.5 mg is administered once daily. Prophylactic doses of 40 mg or 2.5 mg once daily of enoxaparin or fondaparinux, respectively are only used in patients, who are simultaneously treated with dual antiplatelet therapy due to coronary artery disease [72,73].

In addition to that, the use of novel oral anticoagulation agents and, especially, apixaban has been recently proposed as an alternative and more convenient option to subcutaneous therapy. Nevertheless, its benefit should be further investigated, because the current literature is limited [74].

The restoration of the blood flow of the radial artery can be also interventionally achieved from a proximal part of the forearm, where the pulse is still palpated. In patients in whom the insertion of a 5F sheath and a guide wire is successful, a small channel could be opened and, therefore, depicted with the introduction of contrast medium. More severe and total occlusive lesions could be over-passed with the help of a J-typed guidewire and, when this method fails, the thrombi from the radial artery may be aspirated via a balloon dilation and drilling technique [68].

Of note, retrograde recanalization can be also pursued in our era through dTRA. The most popular method that has been proposed lately is the aspiration of the thrombus directly through the inducer sheath, or through an aspiration catheter. Furthermore, promising results have been retrieved by the conduct of balloon angioplasty through the dTRA, or the transcatheter administration of thrombolytic therapy [64,75].

These techniques are considered safe for major events and vascular complications, as only minimal hematomas (EASY Class I-II) and one case of artery perforation have been described. Finally, the retrograde reopening of the radial artery offers a long term patency result—after one, three, six and nine months from the intervention—in most of the investigated cohorts [76].

Nonetheless, these invasive management strategies to restore blood flow cannot be routinely utilized, and the use of this recanalized radial artery as a graft for coronary bypass surgery or for arteriovenous fistula formation could be a risky choice, as this subject is not sufficiently investigated.

## 9. Conclusions

RAO remains the major complication of transradial catheterization. RAO incidence can be limited by the introduction of certain preventive strategies. Adequate anticoagulation, the use of ultrasound before the puncture of the artery, patent hemostasis, and shorter hemostasis time are the main preventive means that the interventionists should consider. Additionally, it should be noted that the introduction of the distal radial access as the main catheterization approach appears to be revolutionary, as far as limiting the rates of RAO incidence is concerned.

## Figures and Tables

**Table 1:** RAO-related risk factors [3,5,6,18,19,20,21,22,23,24,25,26,27].

Patient Related Risk Factors		Procedure Related Risk Factors
Modifiable	Non Modifiable	Modifiable
Diabetes Mellitus	Age	Sheath to Artery Mismatch
Dyslipidemia	Female Sex	Anticoagulation
Peripheral Arterial Disease	Radial Artery Diameter	Multiple Punctures
Multivessel CAD	Low BMI	Long Procedural Time
Reduced Renal Function	Previous Catheterization	Use of Multiple Catheters
		Long Hemostasis Time
		Occlusive Hemostasis
		Radial Artery Spasm

## Data Availability

Not applicable.
